# Comparison between enzyme‐linked immunospot assay and intracellular cytokine flow cytometry assays for the evaluation of T cell response to SARS‐CoV‐2 after symptomatic COVID‐19

**DOI:** 10.1002/iid3.617

**Published:** 2022-09-07

**Authors:** Juliette Villemonteix, Laure Cohen, Amélie Guihot, Valérie Guérin, Clémentine Moulin, Marion Caseris, Agnès Carol, Stéphane Bonacorsi, Guislaine Carcelain

**Affiliations:** ^1^ Laboratory of Immunology, Robert Debré Hospital, APHP Université de Paris Paris France; ^2^ General Pediatrics and Infectious Diseases Department Robert Debré Hospital, APHP Paris France; ^3^ Laboratory of Immunology, Pitié‐Salpétrière Hospital, APHP Paris Sorbonne Université Paris France; ^4^ Laboratory of Immunology Robert Debré Hospital, APHP Paris France; ^5^ Laboratory of Microbiology Robert Debré Hospital, APHP Paris France; ^6^ Laboratory of Microbiology, Robert Debré Hospital, APHP Université Paris Cité Paris France; ^7^ Unité Inserm U976 Université Paris Cité Paris France

**Keywords:** cellular immunity, ELISpot assay, intracellular cytokine staining assay, memory T cells, paucisymptomatic COVID‐19, SARS‐CoV‐2

## Abstract

**Introduction:**

Evaluation of different cell‐based assays for the study of adaptive immune responses against SARS‐CoV‐2 is crucial for studying long‐term and vaccine‐induced immunity.

**Methods:**

Enzyme‐linked immunospot assay (ELISpot) and intracellular cytokine staining (ICS) using peptide pools spanning the spike protein and nucleoprotein of SARS‐CoV‐2 were performed in 25 patients who recovered from paucisymptomatic (*n* = 19) or severe COVID‐19 (*n* = 6).

**Results:**

The proportion of paucisymptomatic patients with detectable SARS‐CoV‐2 T cells was low, as only 44% exhibit a positive T cell response with the ICS and 67% with the ELISpot. The magnitude of SARS‐CoV‐2 T cell responses was low, both with ICS (median at 0.12% among total T cells) and ELISpot (median at 61 SFCs/million peripheral blood mononuclear cells [PBMC]) assays. Moreover, T cell responses in paucisymptomatic patients seemed lower than among patients with severe disease. In the paucisymptomatic patients, the two assays were well correlated with 76% of concordant responses and a Cohen's kappa of 55. Furthermore, in four patients SARS‐CoV‐2 T cells were detected by ELISpot but not with ICS. Short‐term culture could improve the detection of specific T cells.

**Conclusions:**

In patients who recovered from paucisymptomatic COVID‐19, the proportion of detectable anti‐SARS‐CoV‐2 responses and their magnitude seemed lower than in patients with more severe symptoms. The ELISpot appeared to be more sensitive than the ICS assay. Short‐term culture revealed that paucisymptomatic patients had nonetheless few SARS‐CoV‐2 T cells at a very low rate in peripheral blood. These data indicate that various ex‐vivo assays may lead to different conclusions about the presence or absence of SARS‐CoV‐2 T cell immunity.

## Graphical abstract

Evaluation of different cell‐based assays for the study of adaptive immune responses against SARS‐CoV‐2 is crucial for studying long‐term and vaccine‐induced immunity. The ELISpot shows a better sensitivity than ICS. Short‐term culture reveals that paucisymptomatic patients have nonetheless few SARS‐CoV‐2 T cells at a very low rate in peripheral blood and prompt us to be cautious on the conclusion of SARS‐CoV‐2 cellular immunity with classical assays.

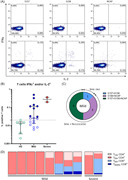

## 1 INTRODUCTION

Since the outbreak of the new coronavirus disease at the end of 2019 (COVID‐19),^1^, ^2^, ^3^ the understanding of the immune response to SARS‐CoV‐2 has become crucial to improve therapeutic interventions and vaccine design. Indeed, while the majority of the COVID‐19 infections are mild, a nonnegligible proportion of patients develop severe illness for which the host immune response seems to be partly involved in the pathogenesis^2^, ^4^, ^5^, ^6^ and reviewed in.^7^ Looking at the disease progression, it is interesting to note that worsening of symptoms usually occurs 7–12 days after symptoms onset^2^, ^8^ which coincides with the natural start‐up of adaptative immunity, especially the expansion of specific T cells. This raises the hypothesis that cellular immunity, whether overactive or too poor in severe cases, contributes to COVID‐19 mortality.^6^, ^9^, ^10^, ^11^ The vaccination campaign is currently in progress, but it is still unclear whether long‐lasting protective immunity can be achieved. Therefore, the study of virus induced immunity is crucial to compare the kinetic of both humoral and cellular responses and may help improve vaccination strategies.

Several studies to date have analyzed SARS‐CoV‐2 specific T cell responses^12^, ^13^, ^14^, ^15^ mostly in severe cases and/or hospitalized patients. Peng et al found that the majority of hospitalized patients exhibited SARS‐CoV‐2 specific T cell responses, with higher frequencies in severe cases compared to mild cases. Grifoni et al. identified T cell responses in almost 100% of COVID‐19 convalescent patients but also in 40%–60% of unexposed individuals suggesting cross‐reactivity on the tested antigens.

The enzyme‐linked immunospot (ELISpot) assay and the intracellular cytokine staining in flow cytometry (intracellular cytokine staining [ICS]) assay are easy to implement and highly sensitive methods for the detection and quantification of cytokine production by antigen‐specific T cells. However, each assay has advantages and disadvantages. A comparison of the data obtained by the two methods was carried out for many viral specificities and the findings show that the results obtained with ELISpot and ICS are not always equivalent.

In this study, we compared the ELISpot and intracellular cytokine flow cytometry assays using specific peptides spanning the full spike protein and nucleoprotein of SARS‐CoV‐2 in patients who recovered from paucisymptomatic or severe COVID‐19. The cytometry analysis also allowed us to determine the state of differentiation of the memory‐specific T cells. Finally, as the proportion of SARS‐CoV‐2 responder was low among our patients, we attempted to increase the sensitivity of T cell detection using in vitro short‐term stimulation.

## 2 MATERIALS AND METHODS

### 2.1 Blood samples

Blood samples (5 ml venous blood lithium heparin) of COVID‐19 mild cases (*n* = 19) were obtained at Robert Debré hospital, Paris. Mild cases were recruited among medical staff with a paucisymptomatic COVID‐19 disease in March 2020. Blood samples of severe cases (*n* = 6) were obtained at an intensive care unit of Pitié‐Salpêtrière Hospital, Paris between March and April 2020. Unexposed healthy donor samples were obtained from the Etablissement Français du Sang before December 2019 and stored in the laboratory. All patients had their SARS‐CoV‐2 infection confirmed by a PCR test in an accredited laboratory.

### 2.2 Isolation of peripheral blood mononuclear cells (PBMC)

PBMC were isolated from freshly collected heparinized blood samples using UNI‐SEP Ficoll tubes (Eurobio Scientific). PBMC were immediately cryopreserved in Fetal calf serum (FCS) + 20% Dimethylsulfoxide (DMSO) at −80°C for future analysis.

### 2.3 Anti‐SARS‐CoV‐2 IgG ELISA

Sera were collected in dry tubes and frozen before further analysis. Anti‐SARS‐CoV‐2 ELISA IgG were performed with EUROIMMUN (Lübeck) kits according to manufacturer recommendations.

### 2.4 Peptides

Three peptide pools, for a total of 417 15‐mers peptides overlapping by 11 amino acid residues and spanning the full Spike and Nucleocapsid, PepMix™ SARS‐CoV‐2 (catalog number PM‐WCPV‐NCAP‐1 and PM‐WCPV‐S‐1), were purchased from JPT (Berlin). These pools were named S158 (mix of 158 spike peptides), S157 (mix of 157 spike peptides) for the spike protein and NCAP (102 NCAP peptides) for the nucleocapsid.

### 2.5 Interferon gamma (IFN‐γ) ELISpot

IFN‐γ‐secreting cells were detected using ELISpot kits (Oxford Immunotec, UK) according to manufacturer's protocol. Frozen PBMC were thawed in RMPI medium + 10% FCS + L‐glutamine 4 mM + Pyruvate 1% + Penicillin 20 U/ml + Streptomycine 20 µg/ml (further designated as “complete medium”), and rested at 37°C for at least 4 h before the assay. Medium was changed for AIM‐V (Oxford Immunotec) and PBMC were plated at 250,000 per well—in duplicate when enough cells were thawed—then incubated 18 h with 1 µg/ml of each peptide pool or with PHA (positive control) or with DMSO (negative control). Spots‐forming cells (SFCs) were counted using Bioreader® 6000E system (Serlabo Technologies) and the number of spots was converted into the number of spots per million PBMC. Quality controls included a negative control to assess the spontaneous IFN‐γ release (wells without stimulation) and a positive control to assess cell functionality (well with the mitogenic stimulator phytohaemagglutinin, PHA). In the negative control, an excess of 10 SFCs have been considered as invalid. For the positive control, the spot count has been considered as invalid if below 20 SFCs. Spots from negative control were subtracted in specific peptide wells. A well with stimulated cells was considered as positive if the number of spots was above 10 SFCs per million of PBMC and was at least twice the number of SFC observed in the negative well.

### 2.6 In vitro short‐term stimulation

Pulsed SARS‐CoV‐2 specific T cells were obtained after 10–11 days culture in complete medium + 10% Human AB serum, with the three SARS‐CoV‐2 pooled peptide mixes (S158 + S157 + NCAP) added at D0 at a final concentration of 2 µg/ml and IL‐2 added at a final concentration of 20 UI/ml with fresh medium at D1, D4, and D7. IFN‐γ‐secreting cells were then detected with the same ELISpot assay.

### 2.7 Cytokine production assay

Intracellular staining (ICS) was performed on thawed PBMC, rested for at least 4 h at 37°C, and stimulated with pooled peptides at a final concentration of 1 µg/ml in complete medium for 18 h with Brefeldin‐A (BFA) (10 µg/ml) added after 1 h of stimulation. After stimulation, cells were washed and surface markers PC7‐anti‐CCR7 and ECD‐anti‐CD28 were stained. Cells were then washed, fixed with IntraStain Fixative/Permeabilization reactives and monoclonal antibodies FITC‐anti‐IFN‐γ, PE‐anti‐CD45RA, APC‐anti‐IL‐2, APC‐AF700‐anti‐CD8, APC‐AF750‐anti‐CD3, PB‐anti‐CD4 were added. Negative (with DMSO) and positive (with anti‐CD3) controls were performed for each sample. All reagents were from Beckman Coulter. Samples were acquired on a Navios cytometer and analyzed with Kaluza Software (Beckman Coulter). A minimum of 10^5^ lymphocytes was acquired. Cytokine responses were background subtracted individually before analysis. Samples were considered positive if at least 0.02% of CD4^+^ or CD8^+^ T cells were positive for the studied cytokines and if at least 10 events were recorded per gate.

### 2.8 Statistical analysis

Figures and statistic tests were made with GraphPad Prism (v8.0.2). Correlations are expressed as Spearman's rank correlation values. Statistical comparisons between mild and severe cases were made with a nonparametric unpaired Mann–Whitney test.

The data supporting the findings of this study are available from the authors upon request.

## 3 RESULTS

### 3.1 Patient's characteristics

Twenty‐five SARS‐CoV‐2 patients were recruited during the first epidemic wave in March 2020, including 19 paucisymptomatic cases recruited among medical staff from Robert Debré Hospital and six severe cases hospitalized in ICU of Pitié Salpêtrière Hospital. Six unexposed individuals were used as negative controls. Patient's characteristics are shown in Figure S1A. Age distribution and time between symptoms onset and sampling show a significative difference between mild and severe groups (*p* = .0058, Figure S1B and *p* = .0004, Figure S1C, respectively), the latter being older and their blood sampling closer to the symptoms.

### 3.2 Evaluation of SARS‐CoV‐2 specific T cells responses

**Figure 1:**
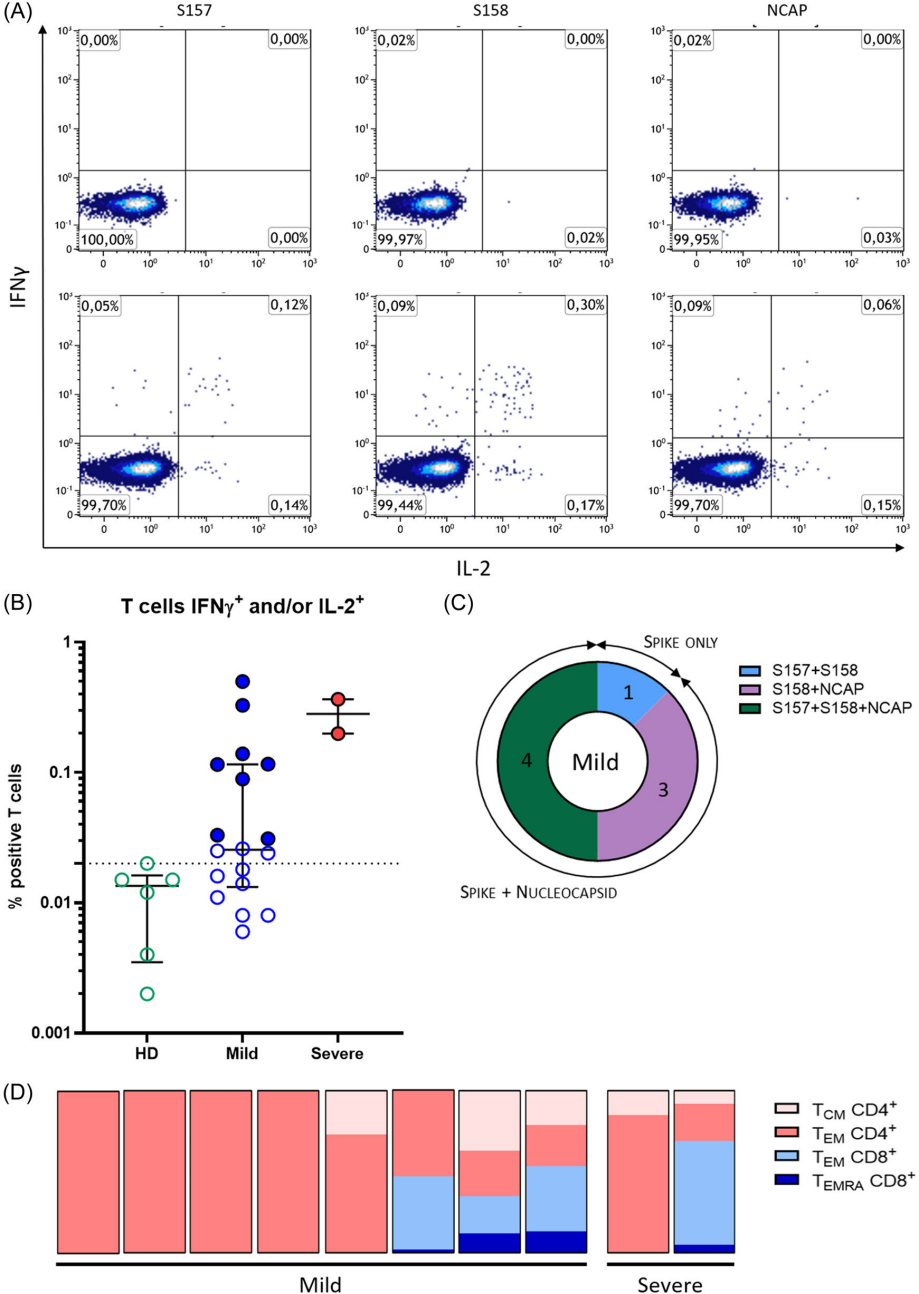
Cytokine release assay for the detection of SARS‐CoV‐2 specific memory T cells. (A) Flow cytometry analysis representing T cells expressing IFN‐γ (*y*‐axis) and IL‐2 (*x*‐axis) after 18 h culture with the respective SARS‐CoV‐2 peptide pools. Percentages are expressed among the T cells. Representative plots of a healthy donor (upper panel) and of a SARS‐CoV‐2 positive patient (lower panel). (B) Proportion of positive T cells for at least one cytokine (IFN‐γ and/or IL‐2) among total T cells upon stimulation with SARS‐CoV‐2 peptide pools for healthy donors (HD) (*n* = 6), mild cases (*n* = 18), and severe cases (*n* = 2). Data are shown as medians with interquartile range. Dotted line represents the positivity threshold. Colored dots represent positive subjects. (C) Repartition among SARS‐CoV‐2 positive subjects of their specific target: spike protein (pools S157 and S158) or nucleoprotein (pool NCAP) or both. (D) Phenotypic characterization of specific SARS‐CoV‐2 T cells. Repartition for each positive patient of the different cellular subpopulations IL‐2^+^ and/or IFN‐γ^+^ upon stimulation with SARS‐CoV‐2 peptide pools: T_CM_ and T_EM_ CD4^+^ (in pink), T_EM_ and T_EMRA_ CD8^+^ (in blue)

**Figure 2:**
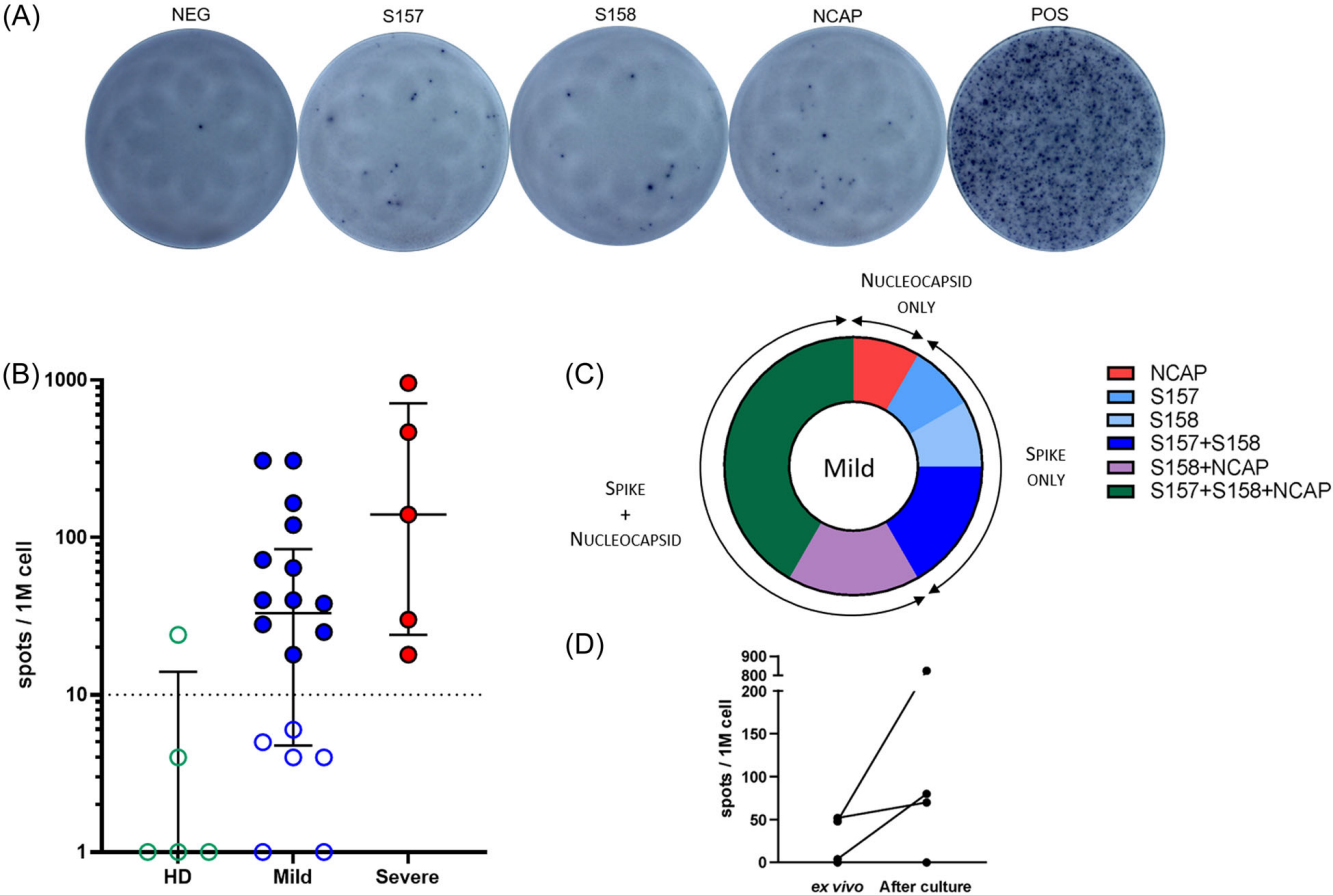
Detection of specific SARS‐CoV‐2 T cells by IFN‐γ Enzyme‐linked immunospot assay (ELISpot) assay. (A) Representative panel of T‐spot assay in a positive patient. From left to right: negative well, S157 peptide pool well, S158 peptide pool well, NCAP peptide pool well, and positive control well. (B) Magnitude of T cell responses against each peptide pool per million peripheral blood mononuclear cells (PBMC) for healthy donors (HD) (*n* = 5), mild cases (*n* = 18), and severe cases (*n* = 5). Data are shown as medians with interquartile ranges. Dotted line representing positivity threshold (10 spots per million PBMC). Colored dots represent positive subjects. (C) Repartition among SARS‐CoV‐2 positive subjects of their specific target: spike protein (pools S157 and S158) or nucleoprotein (pool NCAP) or both. (D) Evolution of T cell responses after culture. PBMC were pulsed with SARS‐CoV‐2 peptides then cultured for 11 days and ELISpot assay were performed at D11 using all peptide pools in one well

The presence of a T cells response was assessed on PBMC in response to three pools of peptides spanning the spike glycoprotein (two pools) and the nucleoprotein of the SARS‐CoV‐2 (one pool). The two assays, intracellular staining by cytometry (ICS) with the quantification of IFN‐γ and/or IL‐2 secreting specific T cells (Figure 1) and enzyme‐linked immunospot (ELISpot) assays with the quantification of IFN‐γ specific T cells (Figure 2), were conducted in parallel. As expected, no T cell response was detected in healthy donors with median at 0.01 (min 0.006–max 0.017) percent of positive cells among T cells for the ICS response (Figure 1B) and at median 0 (min 0–max 8) SFC/10^6^ PBMC for the ELISpot assay (Figure 2B).

While 18 paucisymptomatic patients could be studied by ICS, only two out of six of the severe forms could be studied due to the low number of cells after thawing. As shown in Figure 1, only 8 out of 18 (44%) patients of the mild case group exhibited a positive response with the detection of IFN‐γ and/or IL‐2 secreting T cells in response to SARS‐CoV‐2 peptide pools (Figure 1B). The magnitude of specific T cell responses varied considerably between individuals from 0.03% to 0.5% among T cells with a median of 0.12% (Figure 1B). The majority of the patients of the mild case group (7/8, 88%) had a response against both spike protein and nucleoprotein whereas one patient responded only to the spike protein (Figure 1C). In comparison, the T cell response seemed higher in the limited severe cases analyzed (0.199% and 0.364% of specific T cells).

A specific flow cytometry assay allowed us to assess the phenotype of memory T cells to SARS‐CoV‐2 (see the gating strategy in Figure S2). As shown in Figure 1D, ALL the patients of the mild case group with positive responses had central memory effector CD4 T cells (T_EM_ CD4^+^), three of them having central memory CD4 T cells (T_CM_ CD4^+^). In opposite, only three of these patients had CD8 specific T cells, and they were mostly effector memory CD8 T cells (T_EM_ CD8^+^) although some terminally differentiated T cells that expresses CD45RA (T_EMRA_) were also present (T_EMRA_ CD8^+^). In comparison, these two types of dominant responses, mostly CD4 or CD8, were found in the two severe cases tested although the small number of subjects tested did not allow for any conclusion. We did not detect other subsets such as naïve T cells, T_EMRA_CD4^+^, and T_CM_CD8^+^ among IFN‐γ and/or IL‐2 secreting cells.

Specific T cell responses could be analyzed by IFN‐γ ELISpot in 18 paucisymptomatic patients and five patients with severe case. The percentage of patients of the mild case group with positive responses in ELISpot was high, with 12 out of 18 patients (67%) with detectable SARS‐CoV‐2 specific T cells (Figure 2B). Positive responses ranged from 38 to 308 SFCs/10^6^ PBMC with a median at 61 SFCs/10^6^ PBMC. Only half of the patients of the mild case group (7 out of 12, 58%) displayed a response to both spike and nucleoprotein (Figure 2C). Five out of 12 (42%) patients responded to only one of the two types of proteins, with four responding only to the spike protein (S158 and/or S157) and one only to the nucleoprotein (NCAP) (Figure 2C). In the severe cases group, a T cell response to SARS‐CoV‐2 was detected in all patients (Figure 2B) and all of them to both spike and nucleoprotein (data not shown). Similarly to the ICS assay, responses seemed to be higher in the severe group with a median at 140 (21–957) SFCs/10^6^ PBMC.

### 3.3 Comparison of the two cellular assays

**Figure 3:**
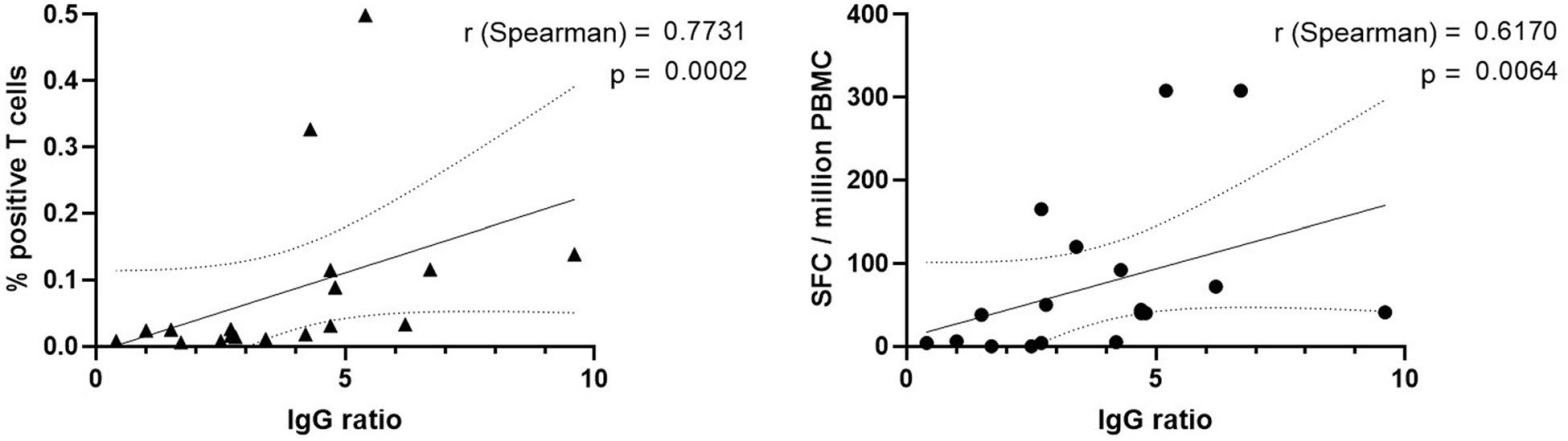
Comparison of humoral and cellular responses against SARS‐CoV‐2. Correlation between cytokine assay—assessed with the total percentage of IFN‐γ^+^ and/or IL‐2^+^ T cells—and the IgG ratio by ELISA (left panel) and between ELISpot assay—assessed with the total SFC count per million PBMC—and the IgG ratio (right panel) in mild cases. The correlation coefficient was calculated with a Spearman's rank test and two‐tailed *p*‐value was then calculated

The relationship between the two cellular assays has been analyzed in 17 paucisymptomatic patients. Overall, the two assays were well correlated as 13 out of 17 (76%) T cell responses were concordant and the Cohen's kappa was 55 (seven positive and six negative responses). However, SARS‐CoV‐2 T cells were detected by ELISpot in four patients (from 50 to 165 SFC/10^6^ PBMC), for whom no specific T cells were detected in ICS. As shown in Figure 3, we also found an overall good correlation between both the proportion of specific T cells quantified by ICS or ELISpot and the IgG titer obtained with an ELISA assay (*r* = .7731, *p* = .0002 and *r* = .6170, *p* = .0064, respectively).

### 3.4 Short‐term culture of SARS‐CoV‐2 specific T cells

The low proportion of patients with paucisymptomatic COVID‐19 cases for which specific memory T cells were detected, led us to question the sensitivity of the tests. Hence, we performed the IFN‐γ ELISpot assay after a 10–11 days culture of PBMC in presence of SARS‐CoV‐2 peptides in four patients whose cells were available. The frequency of specific T cells was increased in three out of four patients as shown in Figure 2D. In two cases, weak positive responses were increased after culture (from 48 to 825 SFCs/10^6^ PBMC and from 52 to 70 SFCs/10^6^ PBMC) and in one case with negative ex vivo response it allowed to detect a positive response of 80 SFCs/10^6^ PBMC.

## 4 DISCUSSION

With the expansion of the SARS‐CoV‐2 outbreak and the consequent development of SARS‐CoV‐2 vaccines, it is important to determine whether exposed or infected people, especially those with very mild or asymptomatic COVID‐19 disease, can develop a cellular immunity.

Using two different cellular assays, we investigated the presence of SARS‐CoV‐2 responsive T cells in paucisymptomatic patients recovered from COVID‐19. We showed low proportion of responsiveness against SARS‐CoV‐2 among these subjects as only 44% have a positive T cell response with ICS and 67% with the ELISpot assay. Those responses are lower than what is reported in the literature.^13^, ^14^, ^15^ We also observed great variation in the proportion of SARS‐CoV‐2 T cell responses and as previously described, this proportion seems higher in severe cases. Of note, this difference could be explained by an earlier evaluation of T cell responses in severe cases in this study. In addition, these characteristics might be related to the fact that studied patients had very few symptoms and few studies focus on this group of patients.^16^ The difference can also be related to the peptides used in the assays. Indeed, we used peptide pools spanning two immunodominant proteins, spike and nucleoprotein.^13^ However, T cell responses have been described against other proteins like the membrane protein or nonstructural ones. Grifoni et al reported that SARS‐CoV2‐ CD4^+^ T cell responses are less dominated by spike protein epitopes than in previous coronavirus infections. Spike accounted for 27% of total responsive CD4^+^ T cells, with membrane (M) and nucleocapsid (N) proteins accounting for 27% and 11%, respectively.^14^ However, this study should be analyzed with caution, as unexposed subjects also had positive responses, raising the possibility of nonspecific activation. Finally, in ICS we used IFN‐γ combined with IL‐2 as a detection cytokine for specific T cells. Study of TNF‐α secretion could have improved the detection of positive T cells.

The analysis of the antigenic repertoire of T cell responses also showed differences between the two cellular assays. A large majority of positive patients in ICS had a broad response to both spike and nucleoprotein, which was not the case for ELISpot assay where less than half of patients responded to both proteins. One explanation is that, the responses of several patients were close to the positive thresholds, making it difficult to analyze their antigenic repertoire.

Regarding the phenotype of the specific SARS‐CoV‐2 T cells, the responses detected in ICS are mostly composed of effector memory CD4 T cells. It is interesting to note that patients with central/effector memory CD8 T cells had a positive SARS‐CoV‐2 PCR with low Ct (data not shown), suggesting that their viral load was higher at the time of infection. In fact, it has been previously shown that viral load is often correlated with strong CD8^+^ frequencies.^17^ Moreover, their IgG levels were slightly over the mean level observed for all patients (data not shown).

The comparison of the two cellular assays showed a good correlation with only four patients showing discordant results between the two tests. For these four patients, SARS‐CoV‐2 T cells were detected by ELISpot while no specific T cells were detected in the ICS, suggesting a better sensitivity of the ELISpot assay. To improve its sensitivity, we performed the ELISpot assay after a short culture of PBMC in presence of SARS‐CoV‐2 peptides. The frequency of specific T cells was increased in three out of four patients tested including one with negative ex vivo response. This observation supports the hypothesis that even with no detectable ex vivo T cell responses, paucisymptomatic patients have few SARS‐CoV‐2 specific T cells in peripheral blood.

The main limitation for this study is the low number of patients tested, especially in the severe cases group. Due to this issue, statistical analysis was not relevant when comparing paucisymptomatic versus severe cases. More severe cases were enrolled but the low number of cells obtained after thawing prevented us from further analysis.

This study shows a significant correlation between the results obtained by ELISpot and ICS assays using SARS‐CoV‐2 peptide pools. However, when measuring low‐level responses, the ELISPOT seems more sensitive, allowing the detection of T cell response in patients with negative results in ICS. The ELISpot assay is also easy to perform, less expensive and more adapted to limited numbers of cells and thus may be relevant to explore large cohort of patients or to analyze the immunization in vaccine trials. Moreover, an in vitro expansion of memory T cells could be of value in case of very low frequency of responder cells.

## CONFLICTS OF INTEREST

The authors declare no conflicts of interest.

## ETHICS STATEMENT

Please note that informed consent was obtained from all subjects and study was approved by local ethics committee. All procedures followed were in accordance with the Helsinki declaration (2000).

## Supporting information

Supporting information.

Supporting information.

## Data Availability

The data supporting the findings of this study are available from the authors upon request.
