# Supplementary figures and images for: Comparison between enzyme‐linked immunospot assay and intracellular cytokine flow cytometry assays for the evaluation of T cell response to SARS‐CoV‐2 after symptomatic COVID‐19
Source: Immun Inflamm Dis. 2022 Sep 7;10(10):e617. doi: 10.1002/iid3.617 (PMC9449588; doi:10.1002/iid3.617)

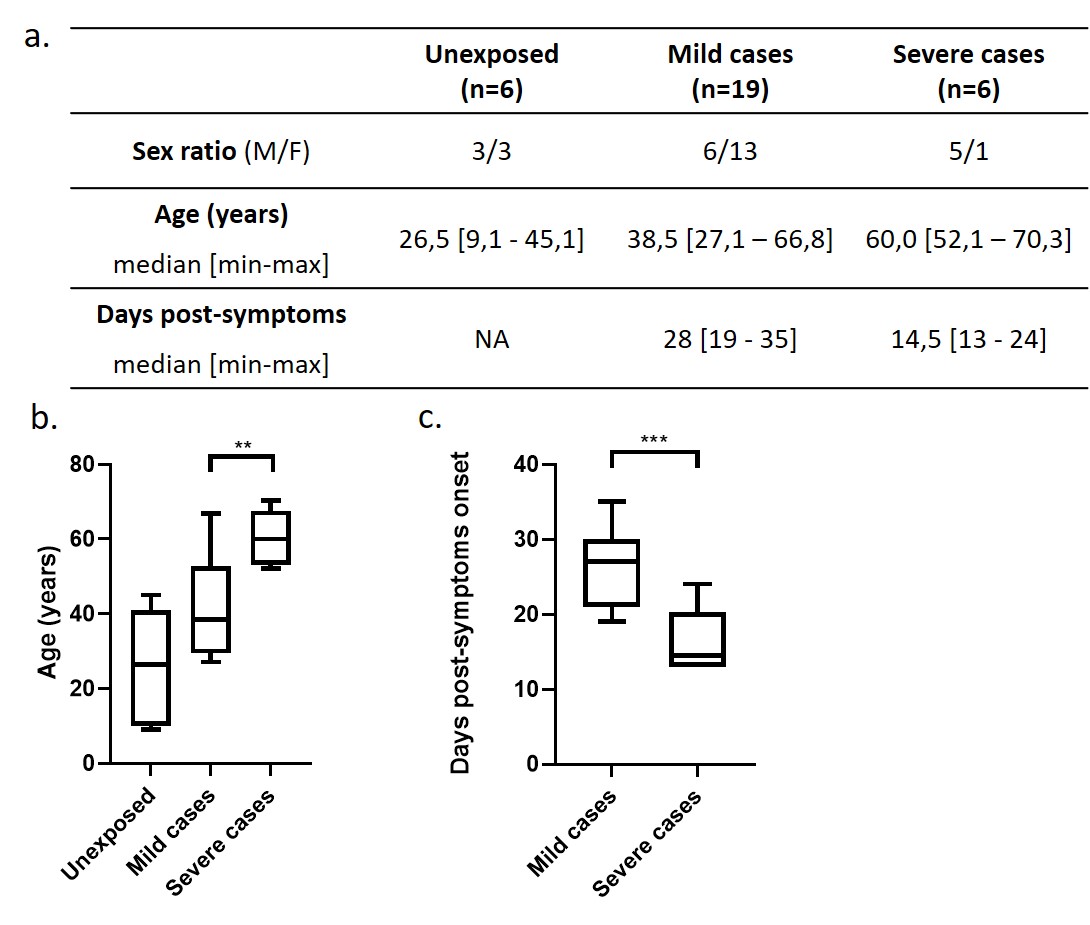

Supplement: Supplementary file 1 — Supporting information. [file IID3-10-0-s001.jpg]

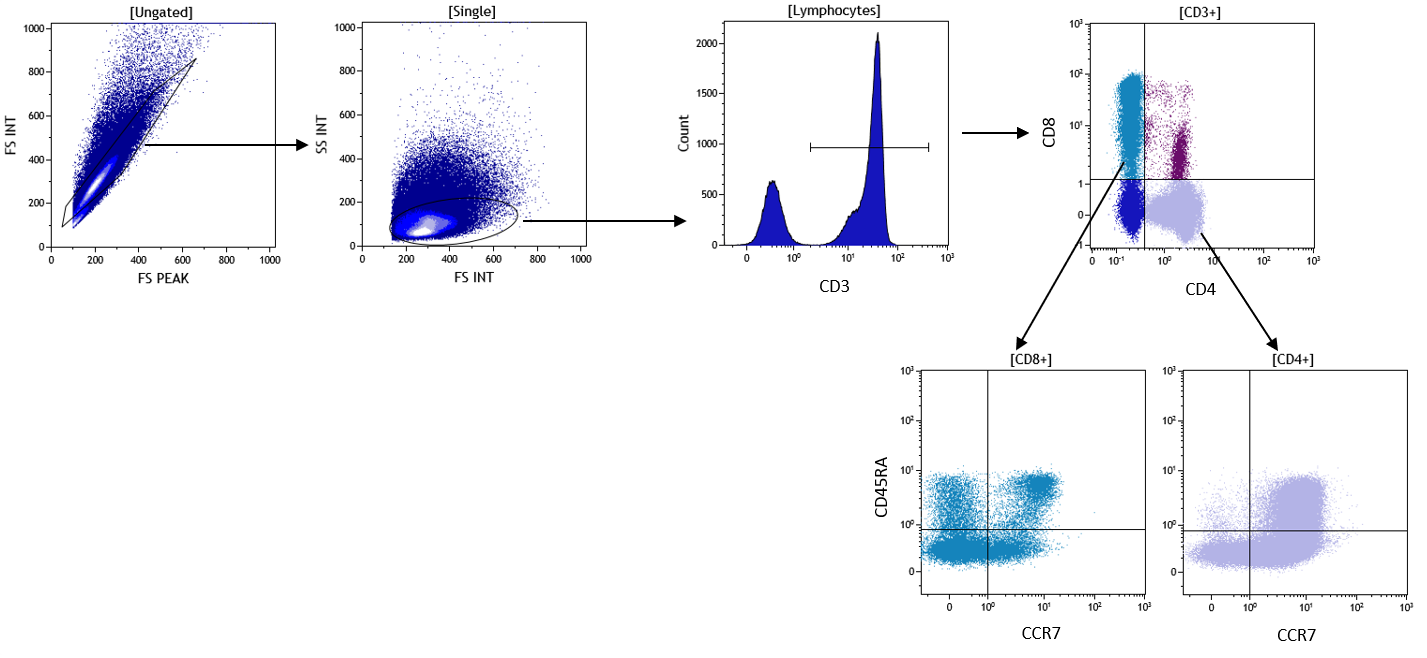

Supplement: Supplementary file 2 — Supporting information. [file IID3-10-0-s002.png]
